# Supplementary material for: Platelet rich Plasma in Achilles Tendon Healing 2 (PATH-2) trial: protocol for a multicentre, participant and assessor-blinded, parallel-group randomised clinical trial comparing platelet-rich plasma (PRP) injection versus placebo injection for Achilles tendon rupture
Source: BMJ Open. 2017 Nov 16;7(11):e018135. doi: 10.1136/bmjopen-2017-018135 (PMC5701990; doi:10.1136/bmjopen-2017-018135)
Supplement: Supplementary file 1 [file bmjopen-2017-018135supp001.pdf]

Supplementary online table

Participating Sites List

Site	Name	PI	Co-PI
01	John Radcliffe Hospital, Oxford	Mr Robert Handley	
02	Leicester Royal Infirmary	Mr Maneesh Bhatia	
03	Musgrove Park Hospital, Taunton	Mr Andrew Kelly	
04	Southmead Hospital, Bristol	Mr Steve Hepple	
05	Princess Royal Hospital, Telford	Mr Michael Carmont	
06	University Hospital of Wales,Cardiff	Mr Paul Hodgson	
07	Royal London Hospital	Mr Nima Heidari	
08	University Hospital Coventry	Mr Jon Young	
09	Warrington & Halton Hospitals	Mr Gareth Stables	
10	Basildon University Hospital	Mr Ravindran Ranjith	
11	Royal Liverpool Hospital	Prof Simon Frostick	Mr Joseph Alsousou
12	Peterborough City Hospital	Mr Jim Carmichael	
13	Morriston Hospital, Swansea	Miss Claire Topliss	Ms Anne-Marie Hutchison
14	University Hospital of Aintree	Mr Lyndon Mason	Mr Joseph Alsousou
15	University Hospital South Manchester	Mr Moez Ballal	
16	Northern General Hospital, Sheffield	Mr Mark Davies	
17	Royal Devon & Exeter Hospital	Mr Adrian Hughes	
18	Leighton Hospital, Crewe	Mr Simon Barnes	Mr Jagan Velpula
19	Royal Surrey Hospital, Guildford	Mr Matthew Solan	
